# A Scorpion Venom-Derived Peptide M6 Endowed with Anti-Aging Ability via Enhanced Antioxidant Activity in Cells, *Caenorhabditis elegans* and Mouse Models

**DOI:** 10.7150/ijbs.133242

**Published:** 2026-05-15

**Authors:** Siyuan Luo, Haixin Qin, Weimin Zuo, Viktor Prypoten, Raymond S. Norton, Yehuda G Assaraf, Hang Fai Kwok

**Affiliations:** 1 Department of Biomedical Sciences, Faculty of Health Sciences, University of Macau, Avenida da Universidade, Taipa, Macau.; 2 Institute of Translational Medicine, Faculty of Health Sciences, University of Macau, Avenida da Universidade, Taipa, Macau.; 3 MoE Frontiers Science Centre for Precision Oncology, University of Macau, Avenida da Universidade, Taipa, Macau.; 4 Cancer Centre, Faculty of Health Sciences, University of Macau, Avenida da Universidade, Taipa, Macau.; 5 Medicinal Chemistry, Monash Institute of Pharmaceutical Sciences, Monash University, Parkville, Australia.; 6 The Fred Wyszkowski Cancer Research Laboratory, Faculty of Biology, Technion-Israel Institute of Technology, Haifa 3200003, Israel.

**Keywords:** Peptide, Anti-aging ability, Antioxidant ability, *C. elegans*

## Abstract

With an aging population and the prevalence of oxidative damage-related ailments, the quest for effective antioxidant and anti-aging agents has emerged as a focal point of biomedical research. Within this domain, peptides have garnered considerable attention for their potential activities. Hence, the current study investigates the antioxidant and anti-aging properties of a scorpion venom-derived peptide M6. We demonstrated that this peptide decreases H_2_O_2_-dependent apoptosis by binding to the TNFR1 receptor, which activates the NF-κB signaling pathway, thereby enhancing the capacity of cells to mitigate oxidative damage induced by H_2_O_2_ treatment. Moreover, M6 prolonged the lifespan of *C. elegans* and enhanced their thermal stress resistance. Further studies demonstrated that M6 inhibits the IIS/PI3K/AKT signaling pathway and activates *daf-16* and *skn-1* (homologous to Nrf-2), thereby enhancing the antioxidant capacity of nematodes and alleviating oxidative damage. Furthermore, M6 also alleviated D-galactose-induced oxidative damage in mice by enhancing the activity of catalase and superoxide dismutase. We also showed that M6 treatment protects liver and kidney tissues from oxidative damage induced by D-galactose. These results highlight the beneficial antioxidant and anti-aging properties of the peptide M6, which appear to be a promising lead for addressing related diseases and mitigating the effects of aging.

## Introduction

Aging is a complex biological process accompanied by gradual decline in cellular functions and impaired tissue homeostasis 1-3. Recent advances in aging research have established a hallmark framework that systematically categorizes the molecular and cellular features of aging, including genomic instability, epigenetic alterations, proteostasis imbalance, deregulated nutrient sensing, mitochondrial dysfunction, and stem cell exhaustion 4, 5. These interconnected hallmarks collectively drive progression of aging and age-related disorders. While the underlying molecular mechanisms remain elusive, mounting evidence points to oxidative stress as a central mediator in aging and associated pathologies, including neurodegenerative disorders, cardiovascular diseases, and metabolic syndromes 6-8.

Reactive oxygen species (ROS), as natural byproducts of cellular metabolism, exhibit dual regulatory roles dependent on concentration gradients. While maintaining critical functions in signal transduction at physiological levels, supraphysiological concentrations of ROS inflict oxidative damage to nucleic acids, proteins, and lipid membranes, ultimately triggering cellular dysfunction and apoptotic pathways 9, 10. Physiological ROS homeostasis is preserved through a dynamic equilibrium between generation and elimination mediated by endogenous antioxidant systems 11-13**.** This redox balance becomes progressively compromised during aging, resulting in cumulative oxidative damage that not only directly impairs biomolecular integrity but also activates senescence-accelerating signaling cascades 14-17.

Cellular defense mechanisms against oxidative stress comprise a two-tiered system: enzymatic components such as superoxide dismutase (SOD) and catalase (CAT) that catalyze ROS detoxification, as well as non-enzymatic molecules such as glutathione that directly neutralize reactive species 18-20. Age-dependent deterioration of these protective systems is exemplified by diminished myocardial antioxidant enzyme activity in senescent rats, which exacerbates cardiac oxidative injury 21. These observations underscore therapeutic strategies targeting ROS scavenging as well as endogenous antioxidant potentiation as viable approaches to mitigate age-related functional decline 22-25. Epidemiological evidence further supports the prophylactic potential of dietary antioxidants (vitamin E, vitamin C, β-carotene) in reducing atherosclerotic risk 26, 27, highlighting the imperative to develop novel compounds with enhanced antioxidant efficacy for aging intervention.

Current antioxidant development encompasses various compounds, including ascorbic acid (vitamin C), α-tocopherol (vitamin E), and polyphenolic derivatives. However, their clinical translation faces significant pharmacological challenges, particularly limited bioavailability—epigallocatechin gallate (EGCG) demonstrates relatively low oral bioavailability in humans 28-30. Even in the case of water-soluble vitamin C, high-dose administration achieves < 50% absorption efficiency 31. Safety concerns further compound these limitations, with documented nephrotoxicity at elevated doses 32, 33. Moreover, it has demonstrated that increased all-cause mortality is associated with chronic high-dose vitamin E intake 34, 35. These constraints, coupled with the inherent complexity of ageing involving multiple pathways, necessitate the development of multi-target antioxidants with improved safety profiles.

Peptide-based therapeutics have emerged as promising therapeutic candidates owing to their intrinsic biochemical advantages. Exhibiting superior target specificity, minimal cytotoxicity, and optimal biocompatibility compared to small-molecule counterparts 36, 37, peptides demonstrate multifunctional capacities encompassing oxidative stress mitigation, modulation of inflammation, and cellular protection 38-40. A peptide derived from bovine casein has been shown to reduce systolic blood pressure significantly and may have potential applications as an anti-hypertensive agent in functional foods or pharmaceuticals 41. Peptides from whey protein hydrolysate have been used to prevent sarcopenia during aging and peptides (from fish collagen) were also used as a commercial product to promote healthy aging and to avoid skin aging 42, 43. The cosmetic sector has successfully commercialized palmitoyl pentapeptide and copper peptide formulations, leveraging their dual collagen synthesis promotion and anti-inflammatory activity 44, 45.

Previously, we demonstrated that a peptide, M6, derived from scorpion venom promotes stem cell proliferation 46. As stem cell exhaustion is one of the hallmarks of aging, we propose that this M6 peptide may have an aging-modulatory effect 5. Here we utilized cell, nematode, and mouse models to conduct *in vitro* and* in vivo* experiments to evaluate the antioxidant and anti-aging activities of the peptide and to explore its mechanism of action. Our current findings not only provide new avenues for the development of peptide antioxidants but also offer potential new strategies for the prevention and treatment of aging-related disorders.

## Methods and Materials

### M6 Preparation

The peptide M6 is under a patent application, hence the sequence will be available upon reasonable request from the corresponding author. M6 (purity: 98.7%) was synthesized from the GenScript Biotech Corporation. For cell and mice works, M6 was dissolved in PBS. For *C. elegans* work, M6 was dissolved in K medium (3.0 g NaCl and 2.36 g KCl in 1 L distilled H_2_O).

### Assessment of the *in vitro* antioxidant ability of peptide M6

#### Cell culture

HUVEC cells were obtained from ATCC and maintained in DMEM containing 10% FBS in 37°C in a 5% CO_2_ atmosphere. When cell confluence attained 80%, the cells were detached by trypsinization and suspended in fresh medium for the following assays.

#### Determination of cell viability

The cell suspension was transferred into a 96-wells plate at a density of 5000 cells/well. After overnight incubation, increasing concentrations of M6 (dissolved in PBS) were added to the plate for another 24 h of incubation. The cells were then exposed to 100 μM H_2_O_2_ for 4 h. Cell viability was then determined by the colorimetric MTT assay.

#### Determination of the ROS level and mitochondrial membrane potential in cells

The cells were pre-treated with M6 for 24 h in 6-well plates. Subsequently, the cells were exposed to 100 μM H_2_O_2_ for 4 hours. Then, the cells were washed three times with PBS. Thereafter, cells were stained using a ROS staining kit (Beyotime, Cat: S0033M) and mitochondrial membrane potential (MMP) detection kit (Beyotime, Cat: C2006) using fluorescence microscopy (EVOS-M7000).

#### Determination of cell apoptosis

The cell suspension was seeded in a 6-well plate and incubated overnight. Then, M6 was then added at various concentrations for 24 h. Subsequently, the cells were exposed to 100 μM H_2_O_2_ for 4 hours. Then, the cells were washed with ice-cold PBS and collected, which were then stained using an apoptosis detection kit (Beyotime, Cat: C1062L) and analyzed using flow cytometry (CytoFLEX Flow Cytometer).

#### Knockdown of TNFR1 expression

The TNFR1 knockdown plasmid, TNFR1-shRNA lentiviral construct, and its matched empty vectors were purchased from Obio Technology Corp., Ltd. (Shanghai. China) and the details of viral vector construction framework were listed in the Supplementary table 1. Cells were seeded in a 6-well plate before lentivirus infection. After attachment, the cells were infected with the corresponding lentiviruses at a suitable multiplicity of infection (MOI) in cell culture medium supplemented with 10 mg/mL polybrene. The culture medium was replaced with fresh medium after 6-8 hours of infection. Following culturing in lentivirus-free medium for 48 hours, cells were selected with puromycin (Sigma, Cat: P9620). Finally, the selected cells were harvested for mRNA and protein analysis to verify the establishment of stable cell lines before expansion.

#### Determination of antioxidant enzymes activity in cells

Cells were collected and instantly frozen in liquid nitrogen and the IP lysis buffer (Beyotime, Cat: P0013) was used to extract cellular proteins. CAT kit (Beyotime, Cat: S0051) and SOD kit (Beyotime, Cat: S0101S) were used to assess the activity of CAT and SOD, respectively.

#### Western blot analysis

Cells were detached from the plate and mixed with RIPA lysis buffer (including Benzonase, protease, and phosphatase inhibitors). Then, the protein concentration of the supernatant was determined by the PierceTM BCA Protein Assay Kit (Thermo Fisher). Equal amounts of protein samples were resolved on 8%~15% SDS-PAGE gradient gels and transferred to a nitrocellulose membrane (NC, 0.22 μm). Then, the NC membrane was blocked in 5% skimmed milk dissolved in 1XTBST for 1 h with gentle shaking at room temperature. Subsequently, primary antibody was added in and the NC was placed on a shaker at 4°C overnight. Then, after removing the primary antibody and washing NC with TBST for 3 times, the second antibody was transferred in, and the NC was incubated for another 1 h before detecting chemiluminescence (Thermo Fisher) on the ChemiDoc Imaging System (Bio-Rad). The details for antibodies can be found in the supplementary Table S2.

#### Molecular docking

The 3D structure of the TNFR1 protein was retrieved from RCSB PDB (BMRB: 30799). Hydrogenation was performed in AutoDock Tools, and docking simulations were conducted using the AutoGrid program to define the binding region. The results were visualized with PyMOL.

### Assessment of the anti-aging and antioxidant activities *in vivo*

#### C. elegans strains and bacteria

Wide-type N2, TJ375 (gpIs1[*hsp-16.2*p::GFP]), CF1553 (muIs84 [(pAD76) *sod-3*p::GFP + rol-6(su1006)]), and CL2166 (dvIs19 [(pAF15)*gst-4*p::GFP::NLS] III), EU1 (*skn-1*[zu67]), GR1307 (*daf-16*(mgDf50)), TJ356 (zIs356[*daf-16*::gfp]), CB1370 (*daf-2*(e1370)), TJ1052 (*age-1*(hx546)), RB759 (*akt-1*(ok525) V.), TG38 (*aak-2*(ok524)), VC204 (*akt-2*(ok393) X.), VC345 (*sgk-1*(ok538) X.) and *E. coli* OP50 were obtained from CGC (Minneapolis, USA). The worms were kept on NGM plates with OP50 as the food source. M6 was dissolved in K medium and mixed with OP50, and then the mixture was spread evenly on the NGM plate. The synchronized worms were obtained by the bleach method 47.

#### Determination of the lifespan

L4 synchronized nematodes were placed on NGM plates and transferred to a new plate every day. The number of dead nematodes was recorded daily until all nematodes had died.

#### Localization of M6 in C. elegans

Wild-type N2 nematodes (L4 stage) were put on NGM plate with a layer of OP50 mixed with FITC or Cy5-labeled M6 (5 μM). After 12 hours of culture, the nematodes were anesthetized, sliced, and the fluorescence intensity was observed under a confocal microscope (Olympus SpinSR10 Spinning Disk Confocal Microscope).

#### Determination of intestinal integrity

Wild-type N2 nematodes (L4 stage) were put on the NGM plate for 72 hours. Then the worms were transferred to a 1.5 mL tube containing OP50 solution and brilliant blue dye for 1 hour. After that, the worms were washed with K medium to remove brilliant blue dye. Finally, these worms were anesthetized, sliced and the integrity of the intestine was observed under a microscope (EVOS-M7000).

#### Determination of the body length and pharynx pumping

Wild-type N2 nematodes (L4 stage) were placed on NGM plates with M6 (5 μM) for 72 h. The pharynx pumping of the worms was observed under a stereomicroscope. Then, the nematodes were anaesthetized and photographed under a microscope (EVOS-M7000). The body length was calculated using Image J.

#### Determination of reproductive ability

Wild-type N2 nematodes (L4 stage) were put on NGM plates with or without M6. The worms were transferred to a new plate every day. The old plate was put in the incubator for 24 h, and the hatched worms were counted.

#### Localization of DAF-16 transcription factor

Synchronized TJ356 nematodes (L1 stage) were placed on NGM plates with M6 (5 μM) for 48 hours. Then nematodes were collected and anesthetized with NaN_3_ (40 mM). The location of DAF-16 was observed under a microscope (EVOS-M7000).

#### Determination of the intake ability

Wild-type N2 nematodes (L1 stage) were distributed to 96-well plates, 20-25 worms per well, then M6 and OP50 were added, along with 25 μM 5-fluorouracil. The plate was placed in an incubator (20°C), and the absorbance value was measured at 600nm every day to detect the consumption of OP50.

Regarding food preference index, L4 stage worms were placed in the middle of a 6-cm plate, with OP50 and a mixture of M6 plus OP50 on both sides. The plate was placed in the incubator for 12 hours and then the number of nematodes on both sides was counted.

#### Determination of the survival rate under thermal stress

The synchronized wild-type N2 nematodes (L4 stage) were treated with M6 for 72 h, then the worms were transferred to 35°C. After 4 and 8 h treatment, the survival rate was recorded.

#### Determination of the ROS level

Wild-type N2 nematodes (L4 stage) were treated with M6 (5 μM) for 72 h. Regarding thermal stress assay, the worms were exposed to 35°C for 3 h. Then, the worms were stained with ROS staining kit (Beyotime, Cat: S0033M) for 1 h in the dark. The worms were then washed with K medium. Subsequently, the nematodes were evenly transferred to 96-well plates (13-15 worms per well), and the fluorescence intensity was read in a microplate reader.

#### Visualization of GST-4, SOD-3 and HSP-16.2

TJ375, CF1553, and CL2166 nematodes (L4 stage) were treated with M6 (5 μM) for 72 h or 3 h at 35°C to induce thermal stress. Then the worms were collected and anesthetized with NaN_3_ (40 mM). The fluorescence intensity was observed with a fluorescence microscope (EVOS-M7000).

#### Determination of gene expression

L4 stage worms were treated with M6 (5 μM) for 72 h. For thermal stress assay, the worms were exposed to 35°C for 3 h. Then worms were also collected and instantly frozen in liquid nitrogen. Next, the nematodes were crushed with a glass rod, and RNA was extracted using RNAeasy™ Animal RNA Isolation Kit with Spin Column (Beyotime, Cat: R0027) and then reverse-transcribed to obtain cDNA (Bio-Rad, Cat: 1708891). Quantitative Real-time PCR (qRT-PCR) was then performed using the SYBR method to detect gene expression according to the Bio-Rad CFX96 instructions. The primer details are listed in Supplementary Table 3.

### Mouse studies

#### Mice maintenance

All animal studies were conducted in accordance with the ethical standards set by the Animal Research Ethics Committee of the University of Macau (Approval No. UMARE-050-2023), with strict adherence to institutional and national regulations. The mice (ICR, male) were housed in a specific pathogen-free (SPF) facility at the University of Macau.

Eight-week-old ICR male mice were housed in separate cages. They were divided into four groups: control group, a D-galactose injury group, a low-dose treatment group, and a high-dose treatment group. In the injury group, mice were divided into low-dose and high-dose treatment groups and intraperitoneally injected with D-galactose (500 mg/kg) daily for 6 weeks. The control group was injected with the same volume of PBS. After the first 3 weeks, the low-dose treatment group and the high-dose treatment group were intraperitoneally injected with different concentrations of M6 (20 mg/kg and 40 mg/kg) for the remaining 3 weeks. Mice's weight was recorded every day. After 6 weeks, the mice were sacrificed. The blood and the organs were collected.

### Determination of the MDA, CAT and SOD in the mice's organs

The organs were collected and instantly frozen in liquid nitrogen. Then the organs were ground, and the Cell lysis buffer for Western and IP (Beyotime, Cat: P0013) was used to lyse the organs. Lipid peroxidation is reflected in the formation of malondialdehyde (MDA). Thus, MDA content was assessed using an MDA kit (Beyotime, Cat: S0131S). The CAT and SOD activities were assessed using CAT kit (Beyotime, Cat: S0051) and SOD kit (Beyotime, Cat: S0101S).

### H&E staining assay

The collected tissues were fixed with paraformaldehyde, dehydrated with different concentrations of ethanol, and then embedded in paraffin. The tissue blocks were then cut into 5 mm slices by a paraffin slicer, fixed on a slide, and subsequently H&E stained after dewaxing and rehydration. The slices were scanned under a microscope (Olympus Research Slide Scanner VS200).

### Data analysis

All experiments were conducted in triplicate. The results obtained were expressed as mean ± SD. Student's t-test was used to detect differences between two groups, and one-way ANOVA was used to detect differences among multiple groups.

## Results

### M6 increases Human Umbilical Vein Endothelial cell (HUVEC) viability under H_2_O_2_

**Figure 1:**
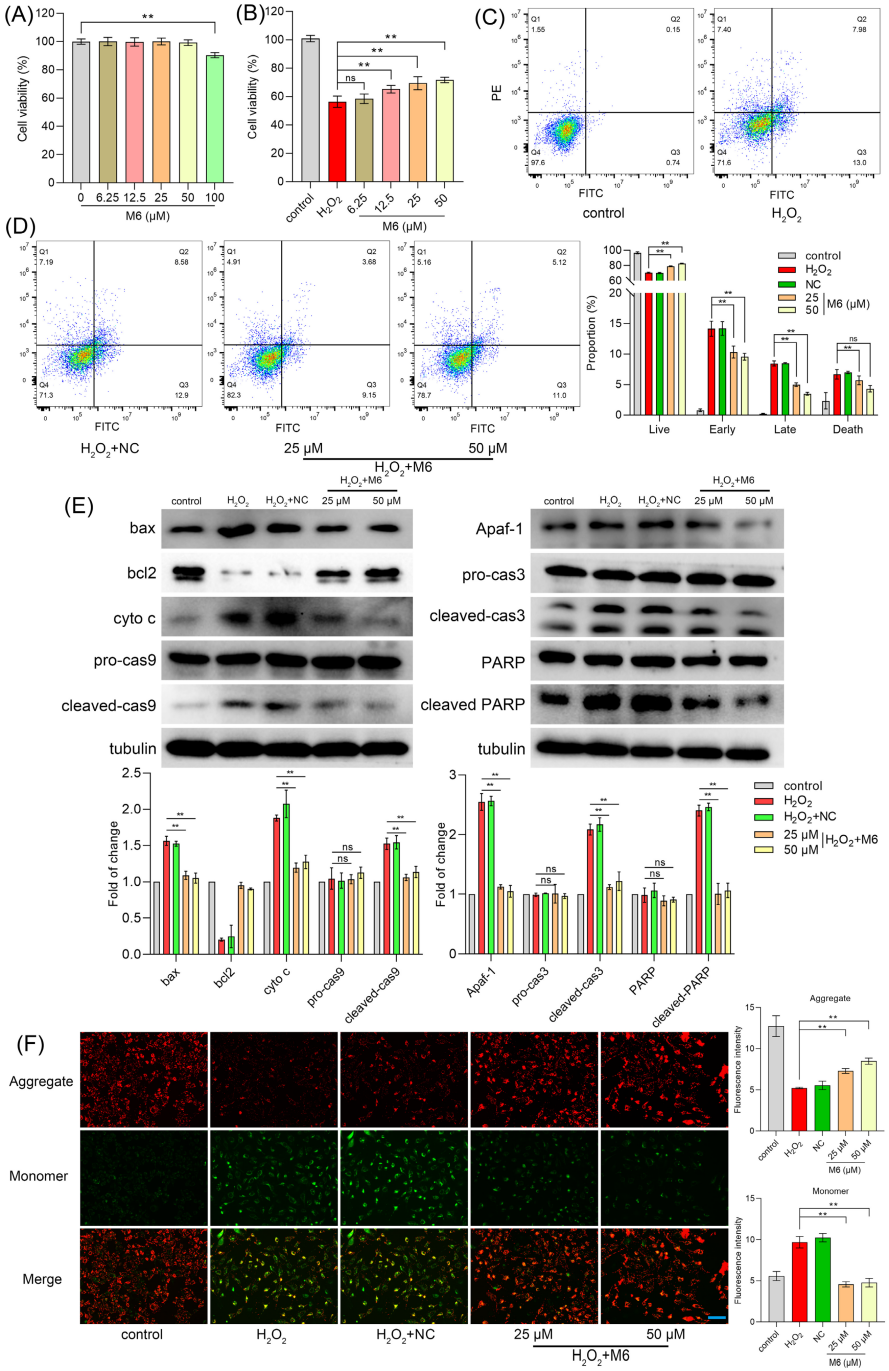
** M6 treatment increases cell ability under H_2_O_2_ treatment. (A)** Cell viability was detected by an MTT assay after cells were exposed to M6 for 24 h. **(B)** Cell viability was detected by the MTT assay after the cells were treated with M6 for 24 h before the cells were exposed to H_2_O_2_ for 4 h. **(C-D)** Cell apoptosis was detected by Annexin V/PI staining assay. **(E)** The expression of apoptosis proteins using WB analysis. **(F)** The MMP assay was employed based on the JC1 staining method. One-way ANOVA method was used to perform statistical analysis. NC - negative control, using the negative peptide (no effect). Ns- not significant, and ** denotes p<0.01. Blue bar, 150 μm.

Cell treatment with increasing concentrations of M6 did not affect cell viability except for at 100 μM, where cell viability was diminished (Figure 1A). Hence, we used concentrations <100 μM for subsequent experiments. To induce exogenous oxidative damage, 100 μM H_2_O_2_ was used as a suitable ROS inducer, known to elicit oxidative stress in cells. Thus, we established an H_2_O_2_-induced oxidative damage model to investigate the *in vitro* antioxidant capabilities of the peptide M6. Remarkably, our findings indicated that after treatment with M6, cell viability was markedly increased in a dose-dependent manner (Figure 1B). Specifically, viability was increased from 56.35% to 71.76% at a concentration of 50 μM M6, revealing the protective effect of this peptide against oxidative damage. For cell assays, a negative control peptide was introduced as the negative control (NC), and the information for NC is recorded in Supplementary Figure 1.

### M6 reduces cell apoptosis induced by H_2_O_2_

Oxidative damage triggered by H_2_O_2_ is known to instigate cell apoptosis and consequently cell demise 48. Figure 1C-D illustrates that after H_2_O_2_ exposure, the percentage of early apoptotic cells reached 14.17%, with late apoptotic cells accounting for 8.46%. Following treatment with 50 μM M6, the proportion of late apoptotic cells decreased markedly to 3.5%, and the number of early apoptotic cells was reduced to 9.56%. Furthermore, cell necrosis was a decline from 6.69% to 4.32%, indicating that M6 treatment reduces cell apoptosis induced by H_2_O_2_. Subsequently, Western blot (WB) analysis was conducted to evaluate the expression of *bona fide* apoptotic proteins. Notably, H_2_O_2_ exposure induced a high expression of apoptotic proteins (Figure 1E), whereas M6 treatment led to a decrease in Bax levels and an increase in Bcl-2 expression, consequently reducing the release of cytochrome C (Figure 1E). These findings were corroborated by JC1 staining assay, in which H_2_O_2_ exposure prompted a transition from JC1 aggregates to a monomer, inducing a decline in mitochondrial membrane potential (MMP) (Figure 1F). In contrast, following cell treatment with M6, the decline in MMP induced by H_2_O_2_ was attenuated. This mitigation was associated with a reduction in the expression of mitochondrial apoptosis pathway proteins, including caspase 9 and Apaf-1 proteins. Furthermore, the levels of caspase 3 and PARP, pivotal proteins in executing apoptosis, were also reduced (Figure 1E), thereby attenuating cell apoptosis.

### M6 activates NF-κB and reduces ROS

**Figure 2:**
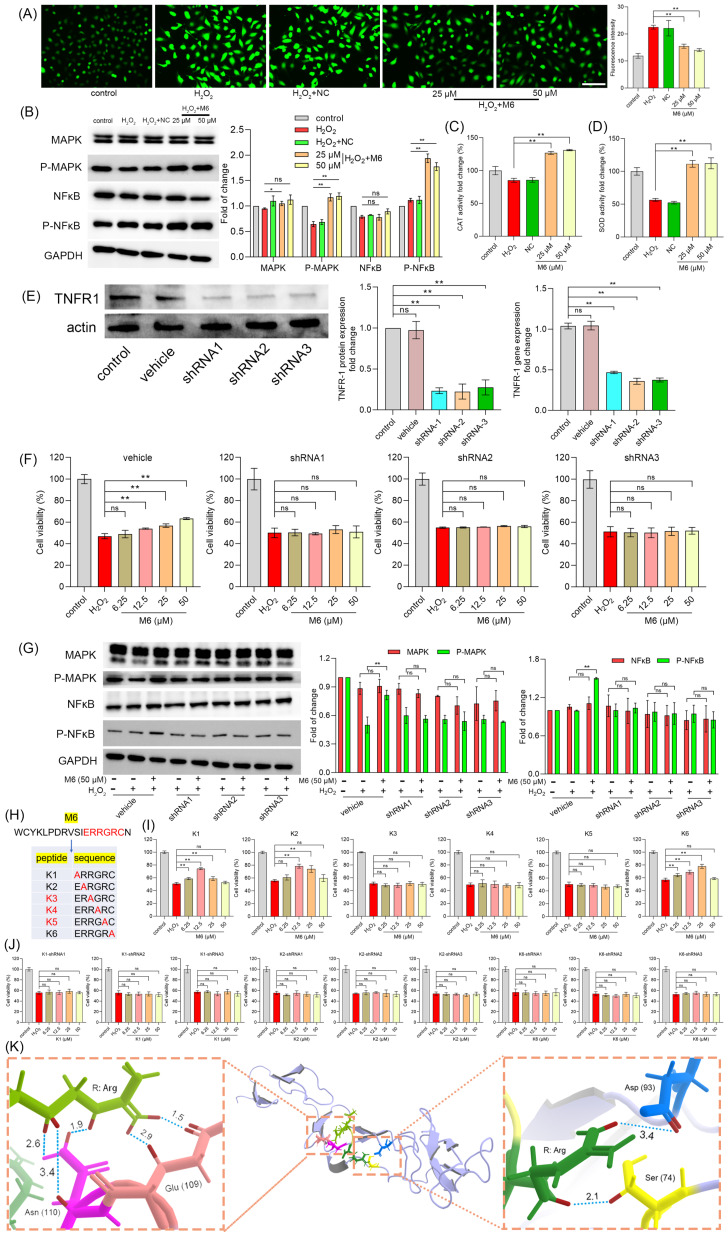
** M6 treatment activates the NF-κB pathway, thereby enhancing the antioxidant ability of cells. (A)** ROS level was detected after the cells were treated with M6 for 24 h before being exposed to H_2_O_2_ for 4 h. **(B)** MAPK and NF-κB levels. **(C-D)** Activity of CAT and SOD in cells after 4 h exposure to H_2_O_2_. **(E)** Expression of TNFR1 protein and TNFR1 gene in transfected cells. **(F)** Cell viability was detected using the MTT assay after these transfected cells were treated with M6 for 24 h before exposure to H_2_O_2_ for 4 h. **(G)** MAPK and NF-κB levels in the transfected cells after exposure to H_2_O_2_. **(H)** The sequences of M6 and the K1-K6 mutant peptides. **(I)** Cell viability of HUVEC cells was detected by MTT assay after the cells were treated with K1-K6 peptides prior to exposure to H_2_O_2_. **(J)** Cell viability of the TNFR1 knockdown cell lines treated with K1, K2 and K6 before exposure to H_2_O_2_. **(K)** Molecular docking between RGR tripeptide and TNFR1. A one-way ANOVA method was performed for statistical analysis. NC means negative control, using the negative peptide (no effect). Ns, not significant, * denotes p<0.05 and ** p<0.01. White bar, 150 μm.

As an exogenous oxidative stressor, H_2_O_2_ induces elevated levels of ROS within cells, leading to a detrimental assault on biological macromolecules, ultimately culminating in cell apoptosis and necrosis 49. Our results showed that M6 mitigated these elevated ROS levels effectively (Figure 2A). Within cells, the antioxidant system plays a pivotal role in regulating oxidation levels, with key defense mechanisms such as the central enzymes CAT and SOD vital in neutralizing free radicals induced by ROS 50. The NF-κB signaling pathway, known for its ability to promote cell survival, encompasses downstream target genes including antioxidant enzymes 51. Figure 2B shows that cell treatment with M6 increased the level of p-MAPK and p-NF-κB. We observed a decline in CAT and SOD activity within the H_2_O_2_-induced injury group, elucidating the heightened ROS content. Following M6 treatment, CAT and SOD activity were restored, contributing to the mitigation of elevated ROS levels (Figure 2C-D). These findings signify that M6 treatment activated the MAPK and NF-κB pathways within cells, thereby enhancing the expression of antioxidant enzyme genes and alleviating the oxidative damage induced by H_2_O_2_.

### TNFR1 receptor is essential for M6 to exert its antioxidant ability

Subsequently, we further evaluated the mechanism through which M6 modulates the NF-κB signaling pathway. In a previous study, we observed that M6 remained extracellular and lacked the ability to penetrate cell membranes 46, leading us to postulate that M6 binds to receptors on the plasma membrane. The TNFR1 receptor serves as the activation receptor for NF-κB, directly influencing the MAPK and NF-κB signaling pathways 52. Hence, we hypothesized that M6 might exert its activity by binding to the TNFR1 receptor. To validate this hypothesis, we engineered a cell line with reduced TNFR1 expression at both the gene and protein levels (Figure 2E). Then, we used M6 to treat these cells to observe the protective effect under H_2_O_2_ condition. Interestingly, we found that M6 treatment failed to increase cell survival (Figure 2F). Moreover, MAPK and NF-κB levels remained unaltered (Figure 2G). These results suggest that M6 cannot exert its antioxidant activity in these cells, indicating that the TNFR1 receptor likely serves as the pivotal receptor through which M6 exerts its antioxidant effects. M6 contains a heparin binding domain bearing the amino acid sequence IERRGRC. Hence, we mutated this domain by converting the glutamate residue to alanine and obtained 6 mutant peptides (Figure 2H). The effects of these 6 peptides were tested using the MTT assay. The results showed that mutant peptides K3, K4, K5 lost their protective activity (Figure 2I). Furthermore, we used TNFR1 knockdown to evaluate the effects of K1, K2 and K6. It was found that cell viability was not improved (Figure 2J). These findings show that TNFR1 is indeed a key receptor for M6 to exert its activity. Furthermore, our results show that the RGR motif in M6 is the key component of its biological activity. We therefore performed molecular docking between RGR and TNFR1. It is evident from Figure 2K that the two Arg residues form contacts with TNFR1 residues Ser 74 and Asp 93 on one side, and Glu 109 & Asn 110 on the other. The hydrogen bond distance between Arg and Asp 93 was 3.4 Å, whereas the distance between Arg and Ser 74 was 2.1 Å, suggesting that Arg can form a stable hydrogen bond with the residues of TNFR1. Moreover, on the other side, another Arg can bind to Glu 109 through two short hydrogen bonds, with distances of 1.5 Å and 2.9 Å, respectively. There were three stable hydrogen bonds between Arg and Asn 110 with distances of 1.9 Å, 2.6 Å and 3.4 Å, which further confirms that the key domain of M6 can form several hydrogen bonds, thereby facilitating M6 binding to TNFR1. It is important to note that the molecular docking analysis presented here is purely computational and predictive. Therefore, future studies using biophysical methods are required to confirm whether RGR truly binds to TNFR1.

### M6 prolongs the lifespan of C. elegans

**Figure 3:**
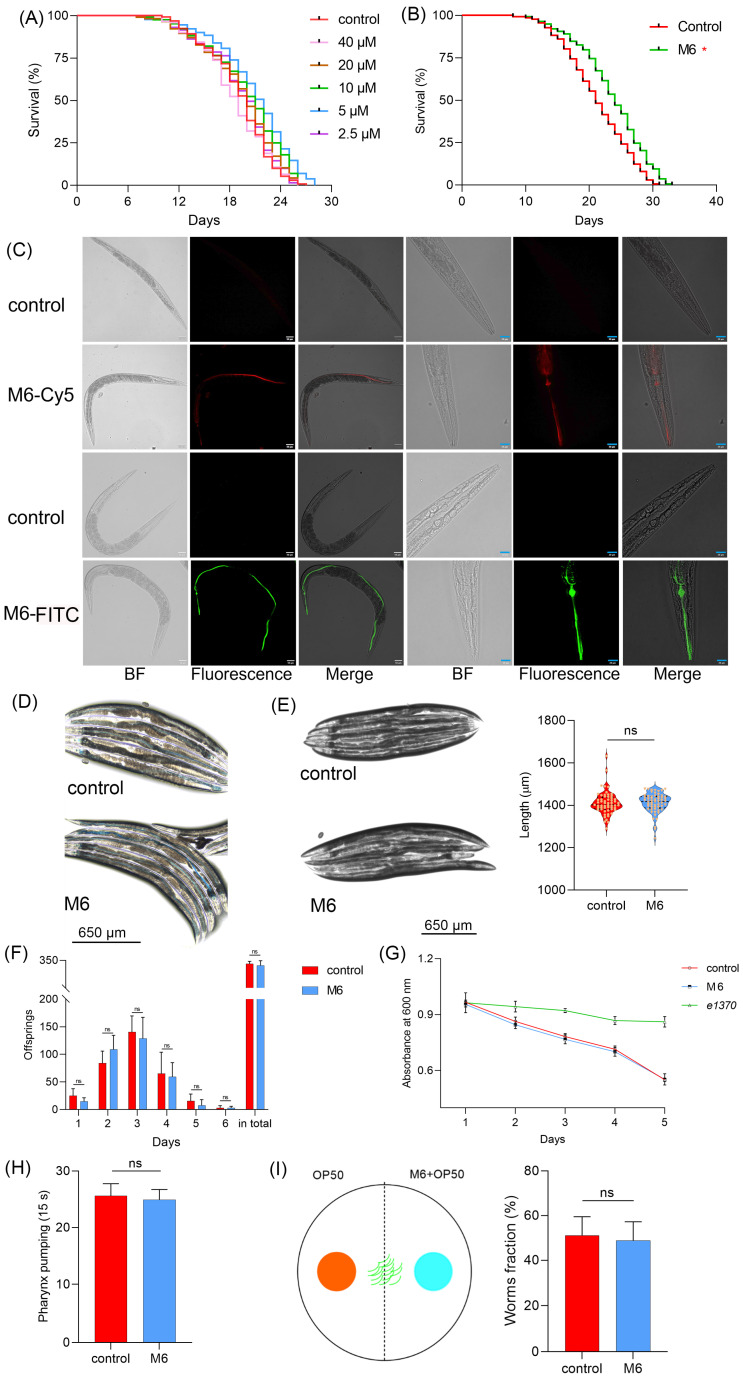
** M6 treatment shows an antiaging effect in *C. elegans*. (A)** Lifespan of N2 worms fed with living OP50 and M6 (n>120). **(B)** Lifespan of N2 worms fed with dead OP50 and M6 (n>120). **(C)** Localization of fluorescent M6 in N2 worms after the worms were fed with M6 for 12 h. **(D)** Intestinal integrity was assessed with a brilliant blue solution mixed with OP50 for 1 h. **(E)** Body length was measured using Image J after the N2 worms were treated with M6 for 3 d (n=57). **(F)** The number of hatched offsprings in N2 worms after treatment with M6 for 6 d (n=10). **(G)** The optical density value of the plate was read at 600 nm for 5 days. **(H)** Pharynx pumping was rated at 15 s after the N2 worms were treated with M6 for 3 d (n=18). **(I)** The worm fraction on each side was recorded (n>120). A Student's T-test was used for statistical analysis of two groups, and Kaplan-Meier survival analysis was applied to evaluate survival outcomes. ns -not significant and * denotes p<0.05. White bar, 80 μm; blue bar, 20 μm.

The robust antioxidant effects of M6 were confirmed at the cellular level. A nematode model was utilized to assess the *in vivo* antioxidant and anti-aging properties of M6, enabling the prompt verification of its efficacy *in vivo*. When nematodes were fed M6 in conjunction with live OP50 at a concentration of 5 μM, the average lifespan of the nematodes was extended from 19.0 days to 21.67 days (p<0.05) (Figure 3A). The potential metabolites of OP50, combined with M6, could influence the lifespan of nematodes. To circumvent metabolic influences, we fed nematodes a mixture of M6 with dead OP50, resulting in a similar extension of the nematodes' lifespan (Figure 3B), which conclusively demonstrates that M6 independently prolonged nematodes' lifespans. Subsequently, FITC and Cy-5 fluorescently labeled M6 were fed worms to observe the localization of M6 in *C. elegans*. We found that fluorescent M6 localized in the pharynx and intestines of nematodes, indicating that the peptide was taken up by the nematodes (Figure 3C). To evaluate potential toxic effects, brilliant blue staining was undertaken; retention of the solution within the nematodes' intestines without leakage affirmed intestinal integrity (Figure 3D). Furthermore, examination of the nematodes' body length revealed no significant alterations (Figure 3E), suggesting that the anti-aging effects of M6 are not associated with toxicity in nematodes.

There exist conserved signaling pathways regulating lifespan, such as dietary and reproductive pathways 53-56. We therefore investigated whether M6 impacted lifespan via these pathways. Figure 3F shows no change in daily egg laying or total egg production, suggesting that M6 did not influence the reproductive signaling pathway. Additionally, food clearance assay demonstrated unaltered feeding abilities after M6 treatment (Figure 3G), with no variance in pharyngeal pumping rates (Figure 3H). Furthermore, Figure 3I reveals that M6 did not incite stimulation in nematodes nor altered their dietary preferences. In summary, M6 extended the lifespans of nematodes independently of the dietary and reproductive signaling pathways.

### M6 activates DAF-16

**Figure 4:**
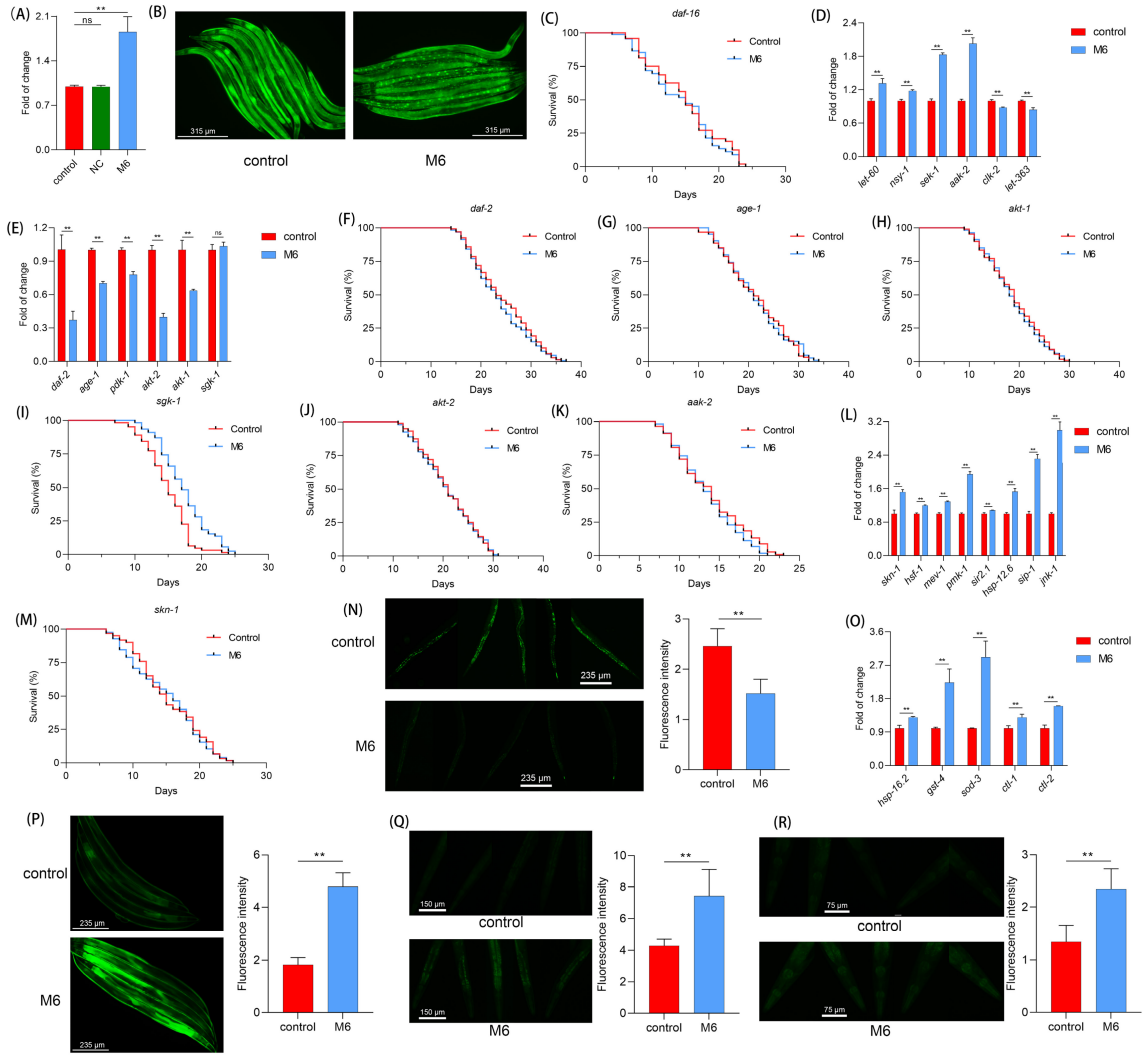
** M6 treatment increases antioxidant capacity via activating *daf-16* in *C. elegans*. (A)** Gene expression of *daf-16* in N2 worms after 3 d of treatment with M6. **(B)** DAF-16 gene expression in the TJ356 strain after the worms were treated with M6 from the L1 to the L4 stage. **(C)** Lifespan of GR1307 mutant with M6 treatment (n>90). **(D)** Gene expression of AMPK/MAPK/mTOR pathways, **(E)** IIS/PI3K/AKT pathways, and **(L)** skn-1/nfr-2 and stress response pathways. **(F-K)** Lifespans of mutants including CB1370 (n>120), TJ1052 (n>100), RB759 (n>100), VC345 (n>110), VC204 (n>100), and TG38 (n>110). **(M)** Lifespan of EU1 mutant treated with M6 (n>110). **(N)** ROS level in N2 worms following treatment with M6 for 3 d from L4 stage. **(O)** Expression of antioxidant genes. **(P-R)** Visualization of GST-4, SOD-3 and HSP-16.2 proteins using CL2166 (n=45), CF1553 (n=45) and TJ375 (n=45) stains treated with M6 for 3 d. NC means negative control, using the negative peptide (no effect). Student's t-test and one-way ANOVA were used to perform statistical analyses for the two- and three-group cases, respectively. Kaplan-Meier method was applied to statistically analyze the lifespan results. ns -not significant and ** denotes p<0.01.

In nematodes,* daf-16* serves as a pivotal factor in regulating lifespan 57, 58. Therefore, we investigated the impact of M6 on *daf-16*. Following M6 treatment, we observed an increase in the expression of *daf-16* (Figure 4A). The TJ356 strain was then used to visualize the subcellular localization of the transcription factor DAF-16. We observed a translocation of the DAF-16 transcription factor from the cytoplasm to the nucleus following M6 treatment of nematodes (Figure 4B), indicating that M6 treatment can activate *daf-16*. Further confirmation utilizing the GR1307 mutant revealed that M6 failed to extend the mutant's lifespan (Figure 4C), suggesting the involvement of *daf-16* in the lifespan extension induced by M6. The expression of the *daf-16* gene is under the regulation of other signaling pathways, such as the insulin signaling pathway 59-61. Subsequent RT-qPCR analysis indicated a reduction in the expression of *daf-2*, *age-1*, *pdk-1*, and *akt-1*, with no change in *sgk-1* expression. Additionally, upregulation of *aak-2*, *let-60*, *nsy-1*, and *sek-1* expression, along with reduced expression of *let-363* and *clk-2*, suggested that M6 might downregulate the mTOR signaling pathway and upregulate the AMPK/MAPK signaling pathway to facilitate *daf-16* activation cooperatively (Figure 4D-E). Experiments using corresponding mutants demonstrated that M6 failed to extend the lifespan of mutants TJ1052, RB759, TG38, VC204, and CB1370, while extending the lifespan of VC345 mutants (Figure 4F-K). These findings indicate that M6 treatment primarily diminished the IIS/PI3K/AKT signaling pathway, consequently activating *daf-16*.

### M6 enhances the antioxidant response in C. elegans

In our cellular investigations, we found that M6 can enhance antioxidant capacity. One of the downstream targets of *daf-16* is linked to antioxidant and stress response abilities 58, 62. The *daf-16* is regulated by multiple pathways. HSF-1 operates in parallel within the IIS pathway to regulate proteostasis 63. SIR-2.1 can deacetylate DAF-16 and modulate its activity 64. And MEV-1 affects mitochondrial ROS production, which in turn influences DAF-16 activation 65. As shown in Figure 4L, the expressions of genes including *hsf-1*, *sir-2.1*, and *mev-1* were increased. Moreover, the skn-1/nrf-2 signaling pathway, highly conserved in mammals, also exists in nematodes, thereby fortifying the body's antioxidant capacity 66. We noted increased expression of *pmk-1* and *skn-1* following M6 treatment (Figure 4L), while M6 failed to extend the lifespan of the EU1 mutant (Figure 4M), indicating the activation of the *skn-1* signaling pathway after M6 treatment. Subsequent assessment of the actual ROS levels in nematodes unveiled a reduction in their levels following M6 treatment (Figure 4N). Further exploration revealed an upsurge in the expression of antioxidant genes in nematodes following M6 treatment (Figure 4O). Utilizing three mutants, CL2166, CF1553, and TJ375, we confirmed elevated expression levels of GST-4, SOD-3, and HSP-16.2 (Figure 4P-R). Collectively, these findings suggest that M6 might boost antioxidant capacity by activating daf-16 and skn-1, thereby diminishing accumulated ROS levels in *C. elegans*, ultimately manifesting antioxidant and anti-aging activities.

#### M6 enhances the thermal tolerance of C. elegans

**Figure 5:**
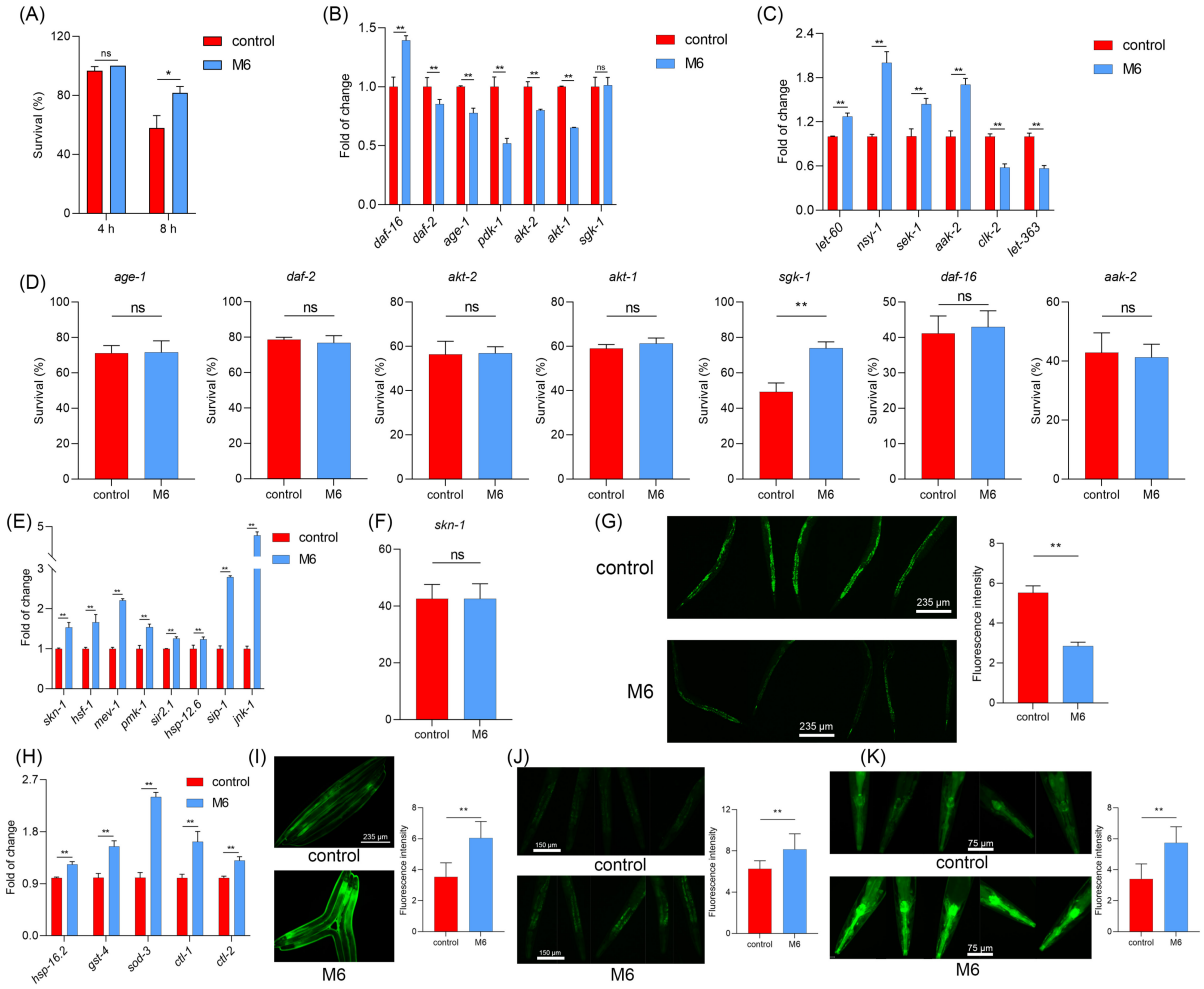
** M6 increases the tolerance of *C. elegans* under thermal stress. (A)** Survival rate of N2 worms after treatment with M6 under thermal stress (35 ^o^C) for 4 h and 8 h (n>70). The expression of genes in N2 worms under thermal stress involving in **(B)** IIS/PI3K/AKT, **(C)** MAPK/AMPK/mTOR and **(E)** skn-1/Nrf-2 and stress response pathways after the worms were exposed to thermal stress (35 ^o^C) for 3 h. **(D)** Survival of mutants under thermal stress for 8 h after 3 d treatment with M6, including TJ1052 (n>60), CB1370 (n>75), VC204 (n>65), RB759 (n>80), VC345 (n>68), GR1307 (n>70), and TG38 (n>90) mutants. **(F)** Survival of EU1 mutant under thermal stress for 8 h after the worms were treated with M6 for 3 d (n>70). **(G)** ROS levels in N2 worms under thermal stress for 3 h. **(H)** Expression of antioxidant genes after the N2 worms were exposed to thermal stress for 3 h. **(I-K)** Visualization of GST-4, SOD-3 and HSP-16.2 in CL2166 (n=45), CF1553 (n=45), and TJ375 (n=45) fluorescence under thermal stress for 3 h after the worms were treated with M6 for 3 d. Student T test was used to perform statistical analysis. ns -not significant, * denotes p<0.05 and ** p<0.01.

Subsequently, we conducted further investigations to determine if M6 treatment could enhance the stress resistance of nematodes, in addition to extending their lifespan. In a thermal stress assay, we observed that nematodes treated with M6 exhibited an improved survival under thermal stress conditions (Figure 5A). Moreover, PCR results revealed that following M6 treatment under thermal stress conditions, nematodes exhibited down-regulation of key genes in the IIS/PI3K/AKT signaling pathway, such as *age-1*, *daf-2*, *akt-1*, and up-regulation of the AMPK/MAPK signaling pathway, including *aak-2*, *nsy-1*, *sek-1* (Figure 5B-C). Further verification of these gene pathways was conducted through analysis of the survival rate of mutants, which demonstrated that following M6 treatment, the survival rate of VC345 mutant was improved, while the other mutants did not exhibit enhanced survival rates (Figure 5D). This effect was associated with the activation of *skn-1* and *daf-16* genes (Figure 5B, E). M6 treatment also led to increased expression of stress-related genes like *mev-1*, *hsf-1*, *pmk-1* (Figure 5E). However, M6 did not enhance the survival of the EU-1 mutant (Figure 5F). These findings collectively suggest that M6 enhances the stress resistance of nematodes by activating the *daf-16* and *skn-1* signaling pathway, akin to its anti-aging pathway.

### M6 improves the antioxidant system in mice

**Figure 6:**
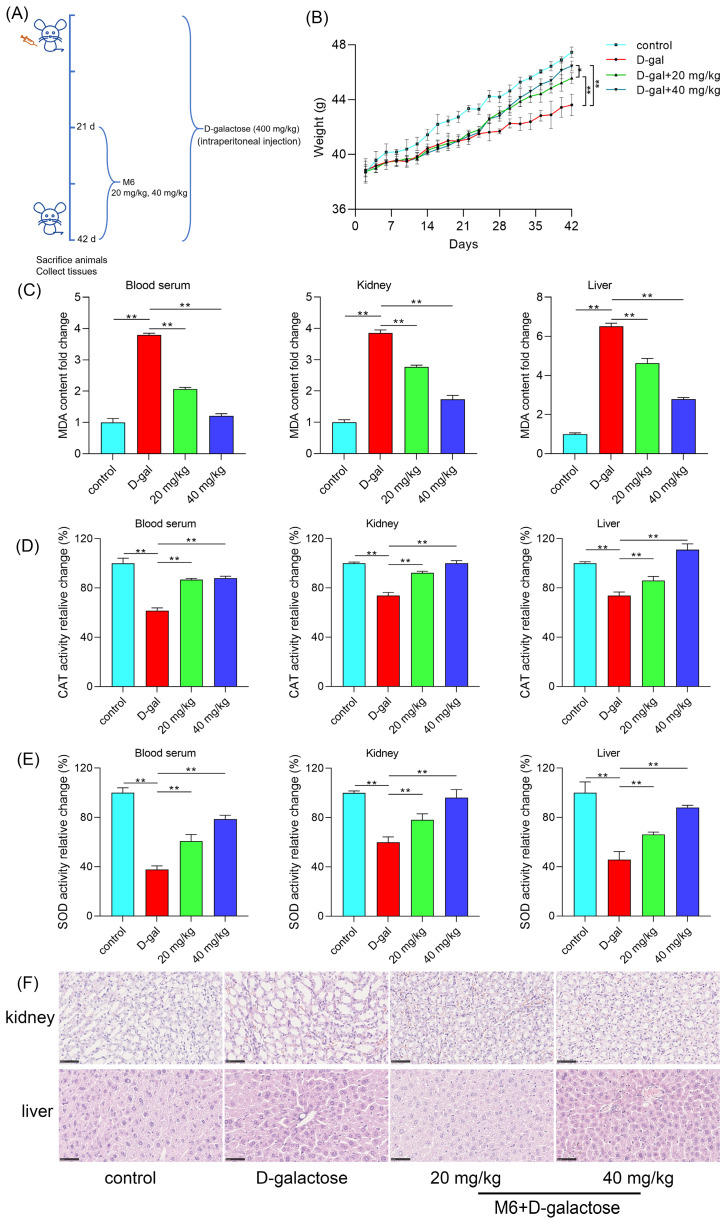
** M6 alleviates the oxidative stress in mice induced by D-galactose. (A)** Timeline schedule of drug administration in mice. **(B)** The weight of mice after injection with D-galactose, M6, and PBS (control group). **(C)** MDA content in serum, kidney and liver. **(D-E)** CAT and SOD activity in serum, kidney and liver. **(F)** The morphological observation of the kidney and the liver using H&E staining. The one-way ANOVA was used for the statistical analysis. * meant p<0.05 and ** meant p<0.01. The black bar is 80 μm.

Nematode experiments offer rapid and dependable results, reinforcing our confidence in the notable antioxidant and anti-aging effects of M6. Therefore, mammalian assays were carried out to corroborate the impact of M6 in mammalian animal models. Prolonged high-dose D-galactose injections disrupt lactose metabolism in mice, resulting in the accumulation of unmetabolized galactose; this accumulation heightens oxidative stress levels, triggering increased free radical production and oxidative stress mediators, ultimately culminating in cellular and tissue damage, thus accelerating the aging process 67. The timeline of drug administration intervention in the mouse experiment conducted in this study is depicted in Figure 6A. Following 21 days of continuous D-galactose injections, mice exhibited weight loss, which was subsequently reversed upon intervention with M6 treatment, as illustrated in Figure 6B. A noteworthy disparity in weight was observed between the M6 intervention group and the D-galactose injury group at the end of the whole process. Elevated levels of malondialdehyde (MDA) were detected in the serum, kidney, and liver of the D-galactose-induced injury group, signifying induced oxidative damage, and reflecting a heightened degree of oxidative stress in the animals. Lipid peroxidation, is reflected in the formation of MDA, and after M6 intervention, a reduction in MDA content in serum and tissues was noted (Figure 6C), indicating the efficacy of M6 treatment in mitigating D-galactose-induced oxidative damage to lipids as well. Furthermore, following M6 treatment, there was an increase in the levels of SOD and CAT compared to the D-galactose injury group (Figure 6D-E), underscoring the potential of M6 treatment to enhance the body's antioxidant capacity and diminish oxidative damage, thereby potentially manifesting antioxidant and anti-aging effects *in vivo*.

### M6 alleviates the injury of the kidney and liver induced by D-galactose

Finally, we employed H&E staining to examine any morphological alterations in liver and kidney tissues that may occur. In the control group, liver cell arrangement exhibited relative regularity, with uniform cell morphology and consistent size and shape of cell nuclei. Conversely, the D-galactose injury group displayed disarrayed liver cell arrangement, devoid of the characteristic radial structure, with abnormalities observed in the morphology of certain liver cell nuclei, such as variations in size and shape, indicative of D-galactose's impact on the normal liver tissue structure (Figure 6F). In the M6-treated group, liver cell arrangement exhibited enhanced regularity compared to the injury group, with cell morphology gradually reverting to normalcy. Furthermore, in the injury group, kidney cells exhibited cavities, excessively large intercellular gaps, and notable variations in the nuclear-cytoplasmic ratio. After M6 treatment, cell morphology gradually normalized, intercellular gaps diminished, and cell morphology returned to a state of equilibrium. These observations collectively underscore that M6 treatment effectively mitigates tissue damage in the aging model, as evidenced by the restoration of tissue morphology in both liver and kidney tissues.

## Discussion

Oxidative stress stands as a pivotal driver of cellular aging and various associated disorders like neurodegenerative diseases, cardiovascular disorders, and cancer 6, 7, 68. The excessive generation of ROS can instigate oxidative damage to DNA, proteins, and lipids, along with mitochondrial impairment, culminating in cellular dysfunction and apoptosis 69-71. Nonetheless, as individuals age, the efficacy of these defense mechanisms gradually diminishes, ushering in the accumulation of oxidative stress and hastening the aging process. Consequently, the pursuit of compounds capable of augmenting antioxidant capacity and retarding aging progression has emerged as a paramount focus in anti-aging research. However, the rate constants and concentrations of conventional antioxidants are often minute, rendering them inadequate to outcompete antioxidant enzymes in neutralizing ROS. Antioxidants primarily furnish preemptive protection against ROS, operating through the neurohormonal system and/or the organism's microbiota to sustain preventive maintenance. Hence, there exists a pressing need to identify potent antioxidants capable of amplifying the activity of antioxidant enzymes, representing a dependable heuristic approach in uncovering the authentic mechanisms underpinning aging and anti-aging therapies 23.

In recent years, natural antioxidants have garnered significant attention owing to their safety and efficacy, particularly antioxidant peptides sourced from food or organisms. These peptides are commonly derived from natural sources such as dairy products, fish, and plant proteins. Their structural and functional resemblance to endogenous peptides in the human body endows them with heightened safety and biocompatibility 36, 37. In contrast to chemically synthesized antioxidants such as butylated hydroxytoluene (BHT) and butyl hydroxyanisole (BHA), natural peptides are less prone to inducing toxic side effects or allergic reactions, rendering them more suitable for prolonged usage 72-74. As a result, the quest for potent and safe antioxidant and anti-aging peptides has emerged as a key focus in contemporary anti-aging research. Further investigation is warranted to delineate the mechanisms of action of peptides and their applicability across diverse models.

**Figure 7:**
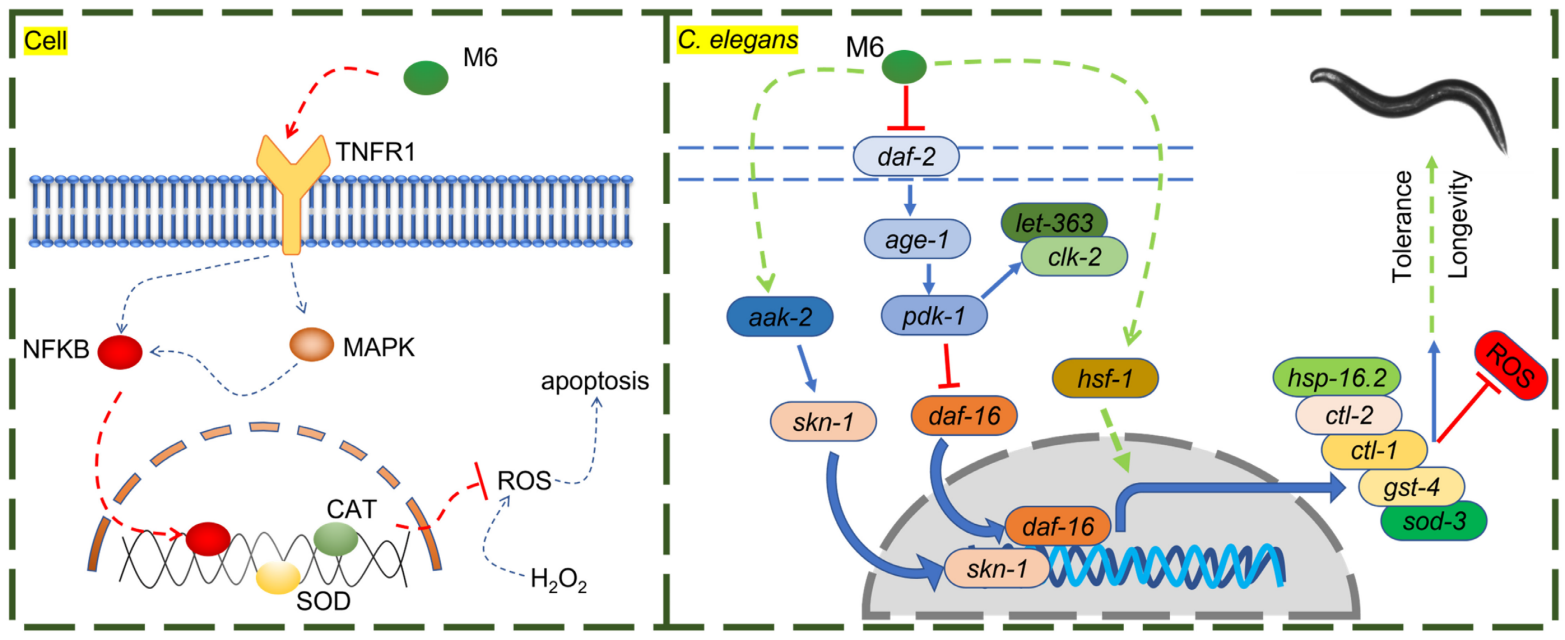
The potential mechanism of action of M6 in cells and *C. elegans*.

The current study comprehensively investigated the antioxidant and anti-aging properties of the peptide M6 using cell cultures, *C. elegans*, and mouse models. In cell experiments, M6 substantially boosted cell survival in an H_2_O_2_ environment and elevated the activities of CAT and SOD by triggering the MAPK and NF-κB signaling pathways. Within cells, the MAPK signaling pathway assumes a central role in cellular stress responses 75-77. The NF-κB signaling pathway, a quintessential antioxidant stress pathway, regulates the expression of multiple antioxidant genes such as SOD, CAT, and glutathione peroxidase 51, 78-80. Moreover, the MAPK signaling pathway can interact with the NF-κB transcription factor to modulate cell survival 81, 82. Activation of the NF-κB signaling pathway can impede apoptosis induced by ROS and the caspase family 83. Our experimental findings demonstrate that M6 can activate MAPK and NF-κB, consequently stimulating the expression of downstream antioxidant systems, such as enhancing CAT and SOD activity, thereby mitigating H_2_O_2_-induced high ROS levels and subsequent apoptosis. These results affirm the pivotal role of MAPK and NF-κB signaling pathways in the cellular-level antioxidant efficacy of M6. It is noteworthy that, upon TNFR1 knockdown, the protective effects of M6 were nullified, suggesting the crucial involvement of TNFR1 in the antioxidant activity of M6. TNFR1, a member of the tumor necrosis factor receptor superfamily, is typically associated with inflammation and apoptosis 84. Upon TNFR1 activation, it can form complex I, which then binds to Fas-Associated protein with Death Domain (FADD) to form complex II, triggering cell apoptosis 85. However, TNFR1 can also avert cell apoptosis and foster cell survival by activating the NF-κB signaling pathway 52, 86. Hence, we postulated that M6 may bind to TNFR1, thereby activating the downstream NF-κB signaling pathway to regulate antioxidant enzyme expression and enhance cellular antioxidant capabilities (Figure 7). This discovery suggests that TNFR1 could serve as the primary receptor for M6 actions, with its antioxidant effects hinging on its interaction with TNFR1.

In the nematode model, M6 significantly extended lifespan and enhanced survival rates under thermal stress conditions. This phenomenon is intricately linked to the inhibition of the insulin signaling pathway (IIS)/PI3K/AKT and the activation of the AMPK/MAPK signaling pathways by M6. The IIS pathway - DAF-2 (ortholog of mammalian INSR/IGF1R), AGE-1 (PI3K), AKT-1/AKT-2 (AKT1/2/3), and DAF-16 (FOXO1/2/3) plays a pivotal role in lifespan regulation, with reduced activity typically associated with lifespan extension 87-89. M6 stimulates the expression of the *daf-16* gene by suppressing the PI3K/AKT signaling pathway. Moreover, M6 can inhibit the mTOR signaling pathway, potentially alleviating cellular metabolic burdens, and retarding the aging process 90, 91. Simultaneously, AMPK serves as a highly conserved metabolic regulatory pathway crucial for cellular energy metabolism. AAK-2 is the worm ortholog of mammalian AMPKα. Its activation can bolster mitochondrial function and autophagy, thereby enhancing cellular stress resilience 92-94. M6 may further augment nematode stress resistance by activating the AMPK/MAPK signaling pathway. *skn-1*, a nematode homolog of *nrf-2*, orchestrates the expression of numerous antioxidant genes, thereby delaying aging in *C. elegans* and mice 95. *hsf-1*, a heat shock factor (the ortholog of mammalian HSF1), is instrumental in regulating the expression of heat shock proteins and preserving protein homeostasis, and is required for lifespan regulation in *C. elegans* and mice 96. Heat shock proteins (HSPs) are considered to be closely associated with thermotolerance and serve as biomarkers of aging 97. M6 promotes the expression of HSP-16.2, which can be partially attributed to the activation of *hsf-1*, contributing to its protective effect under stress. *daf-16*, a FOXO transcription factor homolog and a pivotal gene within the IIS pathway, modulates cell metabolism, stress responses, and lifespan in *C. elegans* and mice 98. In the IIS pathway, DAF-16 (FOXO homolog), and SKN-1 (NRF-2 homolog) act as parallel downstream transcription factors that regulate distinct but overlapping sets of target genes involved in metabolism and oxidative stress resistance 99. The data in this study show that M6 treatment activates both factors, as evidenced by increased expression of *sod-3* (DAF-16 target), and *gst-4* (SKN-1 target). Furthermore, M6 failed to extend lifespan in either the *daf-16* mutant or the *skn-1* mutant (Figure 4C and M), indicating that both transcription factors are required for the pro-longevity effect of M6. This result does not imply a linear relationship between daf-16 and *skn-1*, but rather that M6 acts upstream of both, and loss of either component is sufficient to abolish the benefit. To further confirm the requirement of *daf-16* and *skn-1*, additional experiments using a *daf-16*; *skn-1* double mutant will help determine whether activation of both pathways is also sufficient for full lifespan extension. Through the activation of these genes, M6 could establish a synergistic antioxidant and anti-aging network within nematodes (Figure 7).

The anti-aging efficacy of M6 was also demonstrated in mouse models. M6 substantially decreased the MDA levels in D-galactose-induced aged mice while enhancing the activities of CAT and SOD. MDA is a byproduct of lipid peroxidation 100, 101. The reduction of MDA levels reflects M6's ability to mitigate cell damage caused by oxidative stress induced by D-galactose. In addition, H&E staining results revealed that M6 treatment ameliorated the damage in mice liver and kidney tissues, further affirming the antioxidant and anti-aging properties of M6. Liver and kidney tissues are particularly susceptible to oxidative stress. Factors such as heavy metals and high-fat diets can trigger oxidative stress in these organs, heightening the levels of ROS and MDA within these tissues 102-104. Consequently, our findings suggest that the shielding effect of M6 on these tissues could be linked to its antioxidant capabilities. These outcomes underscore the intimate association between the anti-aging attributes of M6 and its antioxidant ability. It should be noted that the D-galactose model is often used to study brain aging, while the present study primarily focused on peripheral organs (liver and kidney), which are also major targets of aging-related oxidative damage. Future studies with brain are needed to deeply evaluate the potential neuroprotective effects of M6 during aging process.

The experimental results of M6 in cells, *C. elegans*, and mouse models not only confirmed its antioxidant and anti-aging effects but also unveiled the diversity and intricacy of its mechanism of action. The mechanism of action of M6 may entail multi-level regulation: on a molecular scale, through the activation of antioxidant enzymes and genes associated with longevity; on a cellular level, by modulating signaling pathways to mitigate oxidative stress and apoptosis; and on a tissue level, by ameliorating oxidative damage reactions. These multi-tiered mechanisms collectively form the antioxidant and anti-aging network of M6. The experimental outcomes of M6 in cells, *C. elegans*, and mouse models exhibited a high degree of consistency, suggesting that its antioxidant and anti-aging effects are universally applicable across species. These discoveries offer crucial theoretical underpinning for the potential use of peptide M6 in the development of anti-aging pharmaceuticals. While this study has elucidated the diverse mechanisms of action of M6 in antioxidant and anti-aging processes, several pivotal issues necessitate further investigation. For instance, does peptide M6 directly interact with TNFR1? How does it initiate downstream signaling pathways post-binding? These queries could be validated through molecular docking experiments and receptor binding studies. Furthermore, exploring the role of peptide M6 in human disease models, particularly those linked to aging such as neurodegenerative and cardiovascular conditions, warrants additional research.

## Conclusion

This study systematically investigated the molecular mechanisms underlying the antioxidant and anti-aging ability of M6 *in vitro* and *in vivo*. Three key findings emerge: First, at the cellular level, M6 demonstrated significant oxidative stress mitigation by reducing cell apoptosis, activating MAPK and NF-κB pathways to enhance CAT and SOD, thereby lowering the high ROS level. Second, in *C. elegans* models, M6 extended median lifespan while enhancing thermotolerance capacity through conserved longevity pathways, including suppression of the IIS/PI3K/AKT pathways and activation of AMPK/MAPK and Nrf-2 pathways. Third, mouse studies revealed M6 protection against D-galactose-induced senescence, normalizing serum MDA levels, restoration of hepatic SOD activity, and attenuating histopathological damage in renal tissues. These results suggest that M6 can be developed into a promising drug to delay aging and prevent or treat associated diseases.

## Supplementary Material

Supplementary figure and tables.

## Abbreviations

BHT: butylated hydroxytoluene
BHA: butyl hydroxyanisole
ROS: reactive oxygen species
CAT: catalase
SOD: superoxide dismutase
MDA: malondialdehyde
EGCG: epigallocatechin gallate
DMEM: dulbecco's modified eagle medium
PBS: phosphate buffered saline
MTT: thiazolyl blue tetrazolium bromide
MMP: mitochondrial membrane potential
BCA: bicinchoninic acid
TBST: Tris-Borate-Sodium Tween-20
CGC: Caenorhabditis genetics center
NGM: nematode growth medium
H&E: hematoxylin-eosin staining
ANOVA: analysis of variance
