# Supplementary material for: A Scorpion Venom-Derived Peptide M6 Endowed with Anti-Aging Ability via Enhanced Antioxidant Activity in Cells, Caenorhabditis elegans and Mouse Models
Source: Int J Biol Sci. 2026 May 15;22(10):5340–58. doi: 10.7150/ijbs.133242 (PMC13215365; doi:10.7150/ijbs.133242)
Supplement: Supplementary file 1 — Supplementary figure and tables. [file ijbsv22p5340s1.pdf]

A scorpion venom-derived peptide M6 endowed with anti-aging ability via enhanced antioxidant activity in cells, *Caenorhabditis elegans* and mouse models

Siyuan Luo [a, b], *Haixin Qin* [a, b, c, d], *Weimin Zuo* [a, b], *Viktor Prypoten* [e], *Raymond S. Norton* [e], *Yehuda G Assaraf* [f], *Hang Fai Kwok* [a, b, c, d, *]

Supplementary Table S1. Viral vector construction framework

NO.	5’	STEM	Loop	STEM	3’
siRNA1-F	Ccgg	CTTGAAGGAACTACTACTAAG	CTCGAG	CTTAGTAGTAGTTCCTTCAAG	TTTTTTg
siRNA1-R	aattcaaaaaa	CTTGAAGGAACTACTACTAAG	CTCGAG	CTTAGTAGTAGTTCCTTCAAG	
siRNA2-F	Ccgg	TCCCTCCTCTTCATTGGTTTA	CTCGAG	TAAACCAATGAAGAGGAGGGA	TTTTTTg
siRNA2-R	aattcaaaaaa	TCCCTCCTCTTCATTGGTTTA	CTCGAG	TAAACCAATGAAGAGGAGGGA	
siRNA3-F	Ccgg	GCTCTACTCCATTGTTTGTGG	CTCGAG	CCACAAACAATGGAGTAGAGC	TTTTTTg
siRNA3-R	aattcaaaaaa	GCTCTACTCCATTGTTTGTGG	CTCGAG	CCACAAACAATGGAGTAGAGC	
Vehicle-F	Ccgg	TTCTCCGAACGTGTCACGT	TTCAAGAGA	ACGTGACACGTTCGGAGAA	TTTTTTg
Vehicle -R	aattcaaaaaa	TTCTCCGAACGTGTCACGT	TCTCTTGAA	ACGTGACACGTTCGGAGAA	

Supplementary Table S2. The details of antibodies in WB

Antibodies	Company	Cat. number
APAF1	Cell Signaling Technology, Inc.	8969T
PARP	Cell Signaling Technology, Inc.	9542S
MAPK	Cell Signaling Technology, Inc.	4695T
P-MAPK	Cell Signaling Technology, Inc.	4370T
Caspase-9	Cell Signaling Technology, Inc.	9502T
Caspase-3	Cell Signaling Technology, Inc.	9662S
Bax	Cell Signaling Technology, Inc.	2772T
Tubulin	Cell Signaling Technology, Inc.	2144S
Bcl-2	Cell Signaling Technology, Inc.	15071T
Cytoc	Cell Signaling Technology, Inc.	11940T
NFKB	Cell Signaling Technology, Inc.	4249T
P-NFKB	Cell Signaling Technology, Inc.	17366T
Actin	Cell Signaling Technology, Inc.	4967S
GAPDH	Cell Signaling Technology, Inc.	97166
TNFR1	Cell Signaling Technology, Inc.	3736T

Supplementary Table S3. The primers for q-PCR in *C. elegans* and cells

Gene	Forward	Reverse
	C. elegans	
daf-16	TTTCCGTCCCCGAACTCA	ATTCGCCAACCCATGATGG
daf-2	GCGGATACACAGCAAGAATAAC	GAGCCACAAGCACCAGAAC
age-1	CCTGAACCGACTGCCAATC	GTGCTTGACGAGATATGTGTATTG
akt-1	CAAAGCCTAAGGAAGGACAACC	CATGAATCCAACGCTGACGAAC
sgk-1	CACCGACTTTGGGCTCTGTAA	CTTGAGACGAAGTGGCTGGTT
skn-1	AGTGTCGGCGTTCCAGATTTC	GTCGACGAATCTTGCGAATCA
sod-3	CTAAGGATGGTGGAGAACCTTCA	CGCGCTTAATAGTGTCCATCAG
hsp-16.2	CTGCAGAATCTCTCCATCTGAGTC	AGATTCGAAGCAACTGCACC
gst-4	TCCGTCAATTCACTTCTTCCG	AAGAAATCATCACGGGCTGG
hsf-1	TTGACGACGACAAGCTTCCAGT	AAAGCTTGCACCAGAATCATCCC
clk-2	TTCGCTGAGGTTGAACAATCCG	TTGCTGATTGTCATAAGCCATTGC
ctl-1	TTTCAACGGTCGCTGGAGAA	AGTCTGTGGATTGCGCTTCA
aak-2	TCTTCCGCCATCCGCATATC	CCTCTTCATCGGGTCTACGC
pdk-1	ATCGCGGAGACCCATTTGTT	AACAGGAACATTCGGCGTCT
let-363	ACTTGGTTCACTCGTCGGTC	AATTGCGCAACGAACAAGCT
sir-2.1	AAATCTTCCCAGGACAGTTCGTA	ATGGGCAACACGCATAGCA
pmk-1	CGACTCCACGAGAAGGAT	ATATGTACGACGGGCATG
let-60	GAGCAGATTCGGAGGGTAAAG	GTCGACTGATCGAGATGACAAA
ctl-2	TCCCAGATGGGTACCGTCAT	GGTCCGAAGAGGCAAGTTGA
mev-1	CGTCACCAAGTCCGAGGCAAAG	ATGTGGAGCGATTGGGCGATTC
nsy-1	TGCGATGAACTACTACGG	CACCCAAATGACCAAATA
akt-2	AGCACCTTTTGCGGAACACCAG	TCCCCACCAGTCAACACTACGG
sek-1	TGCTCAACGAGCTAGACG	ATGTTCGACGGTTTCACG
hsp-12.6	TGGAGTTGTCAATGTCCTCG	GACTTCAATCTCTTTTGGGAGG
sip-1	CGAGCACGGGTTCAGCAAGAG	CAGCGTGTCCAGCAGAAGTGTG
jnk-1	TGGAACCAGCCAATTCCCAA	TCACAACACTCTGCTCGCAT
act-1	CCAGGAATTGCTGATCGTATGCAGAA	TGGAGAGGGAAGCGAGGATAGA
	Cells	
actin	ATTGGCAATGAGCGGTTCCG	AGGGCAGTGATCTCCTTCTG
TNFR1	TCCAAATGCCGAAAGGAA	TGGAAAAGGTTTTCACTCCAA

Supplementary Figure S1.

The negative peptide (NC) [sequence: WCYKKPDRVSIKEGKRCN] was used as the negative control in this study. The HUVEC cells were treated with NC for 24 h, then the cells were exposed to H_2O_2. The results showed that the cell viability didn't changed.

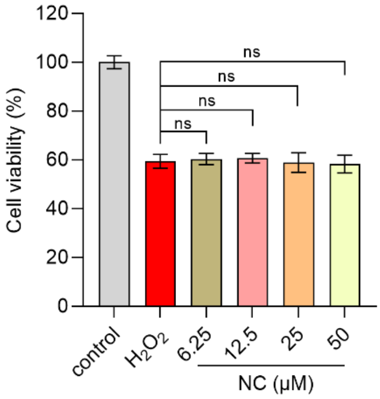

The cell viability of HUVEC under H_2O_2 for 4 h after the cells were treated with NC for 24 h. ns denoted not significance.
